# Insulin Resistance and the Brain–Novel Insights Combining Metabolic Research and Neuroscience

**DOI:** 10.3390/metabo12090780

**Published:** 2022-08-24

**Authors:** Laura Ekblad, Eleni Rebelos

**Affiliations:** 1 Turku PET Centre, University of Turku, PC 20520 Turku, Finland; 2 Institute of Clinical Physiology, National Research Council (CNR), PC 56126 Pisa, Italy

In the last decades, we have been facing an epidemic of obesity and type 2 diabetes (T2D) [1,2]. At the same time, mainly due to an increase in life-expectancy, the prevalence of dementia is increasing rapidly worldwide. Alzheimer’s disease (AD) is the most common cause of dementia. A recent review article concluded that altogether 101 million individuals have either Alzheimer’s dementia or mild cognitive impairment due to AD [3]. While the most important risk factor for AD is old age, there are also acknowledged modifiable risk factors for AD including metabolic risk factors, i.e., obesity, T2D, hypertension, and hypercholesterolemia [4]. Previous studies have also indicated an association between insulin resistance and AD [5,6,7], but it is not clear whether there is any causative relationship between these two conditions.

For obvious reasons, attempts to study further brain metabolism in humans in vivo depend almost exclusively on neuroimaging. Neuroimaging provides the possibility of assessing several brain functions, metabolism, and the structure and accumulation of damaging substances non-invasively within the same subject, and due to its non-invasive nature, it enables examinations of the studied subjects longitudinally and at several stages. Nevertheless, despite the recent, major progress in the neurological field and a better understanding of the role of the brain in the orchestration of whole-body metabolism [8,9,10,11,12,13,14,15], much remains to be investigated in human brain metabolism. 

Based on our previous work on brain metabolism [9,10,11] and studies examining the links between systemic insulin resistance and AD [6,7,16,17], there are two important areas of research that, in our view, require clarification. First, whereas different research groups have coined the term “central insulin resistance” [18] when interpreting their central nervous system-related results in patients with systemic insulin resistance or obesity, an actual clear-cut definition of central insulin resistance using neuroimaging is still missing. Accordingly, the definition of brain insulin resistance has varied depending on the implemented method, i.e., a blunted cerebrocortical insulin effect when magnetoencephalography (MEG) is applied [18], the decreased intranasal insulin-induced suppression of hypothalamic blood flow in functional magnetic resonance imaging (fMRI) [14], and an insulin-induced increase in brain glucose uptake (BGU) in [^18^F]-fluorodeoxyglucose positron emission tomography ([^18^F]-FDG-PET) [10]. Second, although insulin resistance has been demonstrated at the cellular level in post-mortem brain tissues in AD patients [16], no neuroimaging studies have yet examined central insulin resistance in patients with cognitive decline.

We think it would be highly informative to conduct a study where the same subjects would be assessed with at least two of the aforementioned neuroimaging modalities in conjunction with metabolic studies, for instance, using [^18^F]-FDG-PET during a euglycemic hyperinsulinemic clamp and fMRI before and after an intranasal insulin administration. This type of an experimental set-up would help clarify whether an enhanced BGU during hyperinsulinemia and a decreased intranasal insulin-induced suppression of hypothalamic blood flow are associated in the context of systemic insulin resistance. A direct head-to-head comparison on the different modalities that are used as a proxy for central nervous system insulin resistance would establish a more concrete ground for the definition of central insulin resistance with neuroimaging. Furthermore, a combined metabolic and neuroimaging study in patients with cognitive decline (for instance, an assessment of brain glucose metabolism under fasting and insulin clamp conditions) could answer the quandary of whether central nervous system insulin resistance is present in patients with prodromal AD, i.e., mild cognitive impairment due to AD, and would shed light on the question of whether central nervous system insulin resistance can be demonstrated in vivo in these patients.

Another interesting approach to indirectly study the brain is by assessing molecules that originate primarily from the central nervous system but spill over into the blood in quantifiable levels. In recent years, the technology of the single-molecule array (SIMOA) has enabled the adequate quantification of, for example, glial fibrillary acidic protein (GFAP) and neurofilament light chain (NfL) from serum samples [19]. Moreover, the measurement of circulating N-Acetylaspartate (NAA), the second most abundant metabolite of the human brain, is now possible [20,21]. 

In this Special Issue of Metabolites, entitled “*Brain substrate metabolism in health and disease*”, we intend to present an overview of the current knowledge and the latest research and milestones linking obesity, T2D, and insulin resistance to neurologic disorders. Among other topics, we welcome papers focused on (i) novel clinical data on central alterations in the context of metabolic disorders, (ii) intervention studies that aim to improve systemic insulin sensitivity and the brain-related outcomes, (iii) studies on the molecular mechanisms linking insulin resistance to cognitive decline and neurologic disorders, and (iv) methodological advancements in the study of the human brain metabolism in vivo.

## References

[1] Ward Z.J., Bleich S.N., Cradock A.L., Barrett J.L., Giles C.M., Flax C., Long M.W., Gortmaker S.L. (2019). Projected U.S. State-Level Prevalence of Adult Obesity and Severe Obesity. N. Engl. J. Med..

[2] Saeedi P., Petersohn I., Salpea P., Malanda B., Karuranga S., Unwin N., Colagiuri S., Guariguata L., Motala A.A., Ogurtsova K. (2019). Global and Regional Diabetes Prevalence Estimates for 2019 and Projections for 2030 and 2045: Results from the International Diabetes Federation Diabetes Atlas, 9(Th) Edition. Diabetes Res. Clin. Pract..

[3] Gustavsson A., Norton N., Fast T., Frölich L., Georges J., Holzapfel D., Kirabali T., Krolak-Salmon P., Rossini P.M., Ferretti M.T. (2022). Global Estimates on the Number of Persons across the Alzheimer’s Disease Continuum. Alzheimer’s Dement..

[4] Scheltens P., Blennow K., Breteler M.M.B., de Strooper B., Frisoni G.B., Salloway S., Van der Flier W.M. (2016). Alzheimer’s Disease. Lancet.

[5] Matsuzaki T., Sasaki K., Tanizaki Y., Hata J., Fujimi K., Matsui Y., Sekita P.A., Suzuki S.O., Kanba S., Kiyohara Y. (2010). Insulin Resistance Is Associated with the Pathology of Alzheimer Disease the Hisayama Study. Neurology.

[6] Willette A.A., Johnson S.C., Birdsill A.C., Sager M.A., Christian B., Baker L.D., Craft S., Oh J., Statz E., Hermann B.P. (2015). Insulin Resistance Predicts Brain Amyloid Deposition in Late Middle-Aged Adults. Alzheimer’s Dement..

[7] Ekblad L.L., Johansson J., Helin S., Viitanen M., Laine H., Puukka P., Jula A., Rinne J.O. (2018). Midlife Insulin Resistance, APOE Genotype, and Late-Life Brain Amyloid Accumulation. Neurology.

[8] Rebelos E., Nummenmaa L., Dadson P., Latva-Rasku A., Nuutila P. (2021). Brain Insulin Sensitivity Is Linked to Body Fat Distribution-the Positron Emission Tomography Perspective. Eur. J. Nucl. Med. Mol. Imaging.

[9] Rebelos E., Rinne J.O., Nuutila P., Ekblad L.L. (2021). Brain Glucose Metabolism in Health, Obesity, and Cognitive Decline-Does Insulin Have Anything to Do with It? A Narrative Review. J. Clin. Med..

[10] Rebelos E., Immonen H., Bucci M., Hannukainen J.C., Nummenmaa L., Honka M.-J., Soinio M., Salminen P., Ferrannini E., Iozzo P. (2019). Brain Glucose Uptake Is Associated with Endogenous Glucose Production in Obese Patients before and after Bariatric Surgery and Predicts Metabolic Outcome at Follow-Up. Diabetes Obes. Metab..

[11] Rebelos E., Bucci M., Karjalainen T., Oikonen V., Alessandra B., Hannukainen J.C., Virtanen K.A., Latva-Rasku A., Hirvonen J., Heinonen I. (2021). Insulin Resistance Is Associated with Enhanced Brain Glucose Uptake during Euglycemic Hyperinsulinemia: A Large-Scale PET Cohort. Diabetes Care.

[12] Rebelos E., Hirvonen J., Bucci M., Pekkarinen L., Nyman M., Hannukainen J.C., Iozzo P., Salminen P., Nummenmaa L., Ferrannini E. (2020). Brain Free Fatty Acid Uptake Is Elevated in Morbid Obesity, and Is Irreversible 6 Months after Bariatric Surgery: A Positron Emission Tomography Study. Diabetes Obes. Metab..

[13] Boersma G.J., Johansson E., Pereira M.J., Heurling K., Skrtic S., Lau J., Katsogiannos P., Panagiotou G., Lubberink M., Kullberg J. (2018). Altered Glucose Uptake in Muscle, Visceral Adipose Tissue, and Brain Predict Whole-Body Insulin Resistance and May Contribute to the Development of Type 2 Diabetes: A Combined PET/MR Study. Horm. Metab. Res..

[14] Heni M., Wagner R., Kullmann S., Gancheva S., Roden M., Peter A., Stefan N., Preissl H., Häring H.-U., Fritsche A. (2017). Hypothalamic and Striatal Insulin Action Suppresses Endogenous Glucose Production and May Stimulate Glucose uptake during Hyperinsulinemia in Lean but Not in Overweight Men. Diabetes.

[15] Rebelos E., Mari A., Bucci M., Honka M.-J., Hannukainen J.C., Virtanen K.A., Hirvonen J., Nummenmaa L., Heni M., Iozzo P. (2020). Brain Substrate Metabolism and SS-Cell Function in Humans: A Positron Emission Tomography Study. Endocrinol. Diabetes Metab..

[16] Talbot K., Wang H.-Y., Kazi H., Han L.-Y., Bakshi K.P., Stucky A., Fuino R.L., Kawaguchi K.R., Samoyedny A.J., Wilson R.S. (2012). Demonstrated Brain Insulin Resistance in Alzheimer’s Disease Patients Is Associated with IGF-1 Resistance, IRS-1 Dysregulation, and Cognitive Decline. J. Clin. Investig..

[17] Willette A.A., Modanlo N., Kapogiannis D. (2015). Insulin Resistance Predicts Medial Temporal Hypermetabolism in Mild Cognitive Impairment Conversion to Alzheimer Disease. Diabetes.

[18] Tschritter O., Preissl H., Hennige A.M., Stumvoll M., Porubska K., Frost R., Marx H., Klösel B., Lutzenberger W., Birbaumer N. (2006). The Cerebrocortical Response to Hyperinsulinemia Is Reduced in Overweight Humans: A Magnetoencephalographic Study. Proc. Natl. Acad. Sci. USA.

[19] Rebelos E., Rissanen E., Bucci M., Jääskeläinen O., Honka M.-J., Nummenmaa L., Moriconi D., Laurila S., Salminen P., Herukka S.-K. (2022). Circulating Neurofilament Is Linked with Morbid Obesity, Renal Function, and Brain Density. Sci. Rep..

[20] Campi B., Codini S., Daniele G., Marvelli A., Ceccarini G., Santini F., Zucchi R., Ferrannini E., Saba A. (2020). Plasma N-Acetylaspartate: Development and Validation of a Quantitative Assay Based on HPLC-MS-MS and Sample Derivatization. Clin. Chim. Acta.

[21] Rebelos E., Daniele G., Campi B., Saba A., Koskensalo K., Ihalainen J., Saukko E., Nuutila P., Backes W.H., Jansen J.F.A. (2022). Circulating N-Acetylaspartate Does Not Track Brain NAA Concentrations, Cognitive Function or Features of Small Vessel Disease in Humans. Sci. Rep..

